# The Prognostic Impact of Serum Uric Acid on Disease Severity and 5-Year Mortality in Patients With Idiopathic Pulmonary Artery Hypertension

**DOI:** 10.3389/fmed.2022.805415

**Published:** 2022-01-26

**Authors:** Lu Yan, Zhihua Huang, Zhihui Zhao, Qing Zhao, Yi Tang, Yi Zhang, Xin Li, Anqi Duan, Qin Luo, Zhihong Liu

**Affiliations:** ^1^Center for Respiratory and Pulmonary Vascular Disease, National Clinical Research Center for Cardiovascular Diseases, National Center for Cardiovascular Diseases, Department of Cardiology, Fuwai Hospital, Chinese Academy of Medical Sciences and Peking Union Medical College, Beijing, China; ^2^Department of Cardiology, The Clinical Medical Research Center of Heart Failure of Hunan Province, Hunan Provincial People's Hospital, The First Affiliated Hospital of Hunan Normal University, Hunan Normal University, Changsha, China

**Keywords:** hyperuricemia, IPAH, mortality, outcomes, pulmonary hypertension, uric acid

## Abstract

**Background:**

Serum uric acid (UA) has long been identified as a prognostic factor of adverse outcomes in pulmonary hypertension. However, there remains a paucity of evidence on patients with idiopathic pulmonary artery hypertension (IPAH) in the era of targeted drug therapy. This study aims to explore the impact of serum UA levels on the disease severity and mortality in patients with IPAH.

**Methods:**

Consecutive patients diagnosed with IPAH were enrolled, from which UA levels at baseline and the first follow-up were collected. Patients were divided into groups of “hyperuricemia,” which is defined as serum UA level ≥357 μmol/L in women and ≥420 μmol/L in men, and otherwise “normouricemia.” The potential relationship between UA and hemodynamics at right heart catheterization was investigated. Associations between UA and survival were evaluated by Kaplan-Meier analysis and Cox proportional hazard modeling.

**Results:**

Of 207 patients with IPAH, 121 (58.5%) had hyperuricemia. Higher serum UA levels were associated with lower cardiac index (*r* = 0.47, *p* < 0.001) and higher pulmonary vascular resistance (*r* = 0.36, *p* < 0.001). During a median follow-up of 34 months, there were 32 deaths recorded, accounting for a 15.5% mortality rate. Patients with hyperuricemia had a significantly lower survival rate than those with normouricemia (log-rank test, *p* = 0.002). Hyperuricemia at baseline was independently associated with a 2.6-fold increased risk of 5-year death, which was consistent across different subgroups, especially in females and those aged ≥30 years (each *p* < 0.05). Individuals with higher variability in UA had a higher mortality than those with stable UA (log-rank test, *p* = 0.024).

**Conclusions:**

Baseline hyperuricemia and high variability in serum UA at first follow-up were related to a higher rate of 5-year mortality in patients with IPAH. Closely detecting the UA levels may aid in the early recognition of IPAH patients at higher mortality risk.

## Introduction

Pulmonary artery hypertension (PAH) is a devastating condition characterized by progressive remodeling in the pulmonary vasculature, leading to increased pulmonary resistance, right ventricular failure, and even death (1). Despite recent therapeutic advances in PAH-specific management, the mortality of the most common form of PAH—idiopathic pulmonary artery hypertension (IPAH), remains high, particularly in those in the advanced state or resistant to therapies (2, 3). Further, echocardiographic estimates, commonly used to screen for the presence of pulmonary hypertension, are somehow poorly associated with invasive hemodynamic measures at right heart catheterization (RHC) (4). This leaves the clinicians with limited tools to non-invasively assess the presence or the severity of PAH. In light of these challenges, there is an increasing interest in using non-invasive biomarkers for early delineation of disease severity and prognosis during follow-up (4).

Serum uric acid (UA), the end product of purine degradation, has been proposed as a prognostic biomarker in PAH (5, 6) and other cardiovascular diseases (7). Evidence supporting the role of UA in PAH secondary to connective tissue diseases has accumulated over the decades (8, 9). Hyperuricemia has also long been a poor prognostic factor in IPAH, yet before the era of current therapeutic management (10). Also, the relationship between the variability of UA and long-term survival was less well-established until now in patients with IPAH.

Therefore, this study aimed to investigate the associations of serum UA levels and hemodynamic parameters, and analyze the prognostic impact of baseline hyperuricemia and the variability of serum UA on long-term mortality in patients with IPAH.

## Materials and Methods

### Participants

Of the 211 consecutive patients diagnosed with IPAH in our institute between January 2015 and January 2020, three patients were excluded due to the absence of serum UA data, and one was excluded due to lung transplantation. The remaining 207 patients with IPAH were enrolled in the current study. These patients' demographic data [including body mass index (BMI)], World Health Organization (WHO) functional classification, primary laboratory results, serum UA levels, and hemodynamic parameters derived from RHC were gathered and analyzed. The study was performed with the approval of the Fuwai Hospital Ethics Committee (No. 2018-1100). Written informed consent was obtained from each patient included in this study.

### Hemodynamic Studies

Patients underwent diagnostic RHC while in stable condition during hospitalization. A baseline hemodynamic profile comprised of mean pulmonary arterial pressure (mPAP), right atrial pressure (RAP), and pulmonary vascular resistance (PVR) were measured at end-expiration. The cardiac output was calculated using the Fick method, and the cardiac index was obtained by dividing the cardiac output by the body surface area. Following 2015 ESC/ERS guidelines (11), all studied patients were diagnosed with IPAH by RHC according to standard criteria: an mPAP ≥25 mmHg and PVR >3 Wood units at rest in the presence of a normal pulmonary capillary wedge pressure (≤ 15 mmHg), after ruling out connective tissue disease, congenital heart defect, portopulmonary hypertension, human immunodeficiency, heritable, drugs or toxins-induced, left-sided heart disease, pulmonary disease, pulmonary thromboembolism, and other definite causes.

### Serum Uric Acid Measurement

Blood samples for baseline measurements, including serum UA were obtained from a peripheral vein after an overnight fast upon admission. After that, medications including diuretics, cardiac inotropes, and targeted drugs were initiated based on an adequate risk status assessment. This was to reduce their influence on baseline serum UA levels to a large extent. Serum UA levels were determined using the uricase-peroxidase method. Considering the sex difference in serum UA levels, hyperuricemia was defined as baseline UA levels >357 μmol/L in women and 420 μmol/L in men, consistent with previous studies (12). The other primary laboratory results at baseline, including complete blood counts, bilirubin levels, creatinine, lipid profiles, N-terminal pro-B-type natriuretic peptide (NT-proBNP), and C-reactive peptides, were also collected.

To explore the potential impact of variation in UA levels on the prognosis, patients with a second measure of serum UA at the first follow-up within 3 months were retrospectively investigated. Similarly, patients were classified into hyperuricemia and normouricemia groups according to their UA levels at first follow-up. An increase or a decline in UA levels was thereby noted. The serum UA variability (UAV) was the exposure variable of interest. It was defined as the fluctuating amplitude of serum UA levels as compared with the baseline UA level. The median of UAV was 53 μmol/L and was applied to classify patients into a high UAV group (≥53 μmol/L; i.e., a large rise or fall in UA) and a low UAV group (<53 μmol/L; i.e., a slight rise or fall UA).

### Outcome Assessment

The primary outcome of the current study was the rate of 5-year all-cause mortality. Telephone follow-up was used to monitor clinical outcomes every 3–6 months. Patient deaths were identified by medical records or death certificates review. Survival of IPAH patients was estimated from the date of the first catheterization until 5 years or to the date of patient death if that occurred first. One patient who underwent transplantation was removed from the analysis at the time point.

### Statistical Analysis

Continuous variables are presented as the mean ± standard deviations or as the medians (interquartile range) after evaluation of normality. Categorical variables are presented as counts and percentages. Comparisons of variables between the hyperuricemia and normouricemia groups were made by two-tailed independent sample *t*-tests for continuous variables and Pearson's *x*^2^ tests for categorical variables. A non-parametric Mann-Whitney *U*-test was used when data were not normally distributed. Univariate correlation analysis and multiple stepwise linear regression analysis were used to investigate potentially related factors of serum UA levels. Survival curves were derived using the Kaplan-Meier method and were compared using the log-rank test. The independent association of hyperuricemia with mortality was tested by multivariate Cox proportional hazards regression analysis. A *p-*value of <0.05 was required for statistical significance in all cases. Statistical analyses were conducted using *R* statistical version 3.6.3 (*R* Project for Statistical Computing) within RStudio statistical software version 1.1.453.

## Results

### Baseline Characteristics

A total of 207 patients with IPAH (mean age 32.3 ± 10.5 years; 75.8% female; BMI 22.5 ± 3.5 kg/m^2^) were included in this study. The majority was classified as WHO functional class II and III, consisting of 103 and 90 individuals, respectively. The detection levels of serum UA ranged from 121.5 to 929.7 μmol/L. Hyperuricemia was present in 121 patients (58.5%) with IPAH. Approximately 85.5% of participants received targeted therapy for PAH at the index hospitalization. The remaining refused to take mainly due to financial burden, cautions about adverse effects, and intolerability.

Comparisons of demographic and clinical data between hyperuricemia (UA: women ≥357 μmol/L, men ≥420 μmol/L) and normouricemia group (UA: women <357 μmol/L, men <420 μmol/L) were summarized in Table 1. Compared with the normouricemia group, RAP, mPAP, and PVR were significantly higher, while the cardiac index was significantly lower in the hyperuricemia group. Patients with hyperuricemia also had significantly higher white blood cell counts, bilirubin, creatinine, blood urea nitrogen, and notably, NT-proBNP than those with normouricemia (1,510.0 vs. 1,009.4 pg/ml). However, there were no significant differences between the two groups regarding WHO functional classes, laboratory test results such as platelet counts, albumin, lactate dehydrogenase, inflammatory markers, lipid profiles, and medications at index hospitalization.

**Table 1:** Basic characteristics in patients with idiopathic pulmonary artery hypertension.

**Variables**	**Normouricemia**	**Hyperuricemia**	**All**	***P*-value**
	**(*n* = 86)**	**(*n* = 121)**	**(*n* = 207)**	
Age, years	33.4 ± 11.5	31.5 ± 9.6	32.3 ± 10.5	0.196
Female, *n* (%)	72 (83.7)	85 (70.2)	157 (75.8)	0.018
BMI, kg/m^2^	21.9 ± 3.4	23.0 ± 3.5	22.5 ± 3.5	0.025
WHO FC, I/II/III/IV, *n*	6/43/35/2	1/60/55/5	7/103/90/7	0.095
Deaths, *n* (%)	6 (7.0)	26 (21.5)	32 (15.5)	0.003
**Hemodynamic indices**				
RAP, mmHg	4.0 ± 3.6	6.1 ± 4.7	5.2 ± 4.4	<0.001
mPAP, mmHg	54.9 ± 15.5	60.9 ± 16.5	58.4 ± 16.3	0.009
PVR, Wood units	10.4 ± 5.1	14.3 ± 6.6	12.7 ± 6.3	<0.001
CI, ml/min/m^2^	3.2 ± 1.0	2.5 ± 0.7	2.8 ± 0.9	<0.001
**Laboratory tests**				
WBC, × 10^9^/L	6.5 ± 1.7	7.5 ± 3.1	7.1 ± 2.6	0.005
Neutrophils, %	57.5 ± 9.9	56.4 ± 10.8	56.8 ± 10.4	0.472
Platelet, × 10^9^/L	191.4 ± 52.8	184.6 ± 62.2	187.4 ± 58.4	0.414
Albumin, g/L	41.9 ± 5.1	42.0 ± 4.5	42.0 ± 4.7	0.854
TBIL, μmol/L	18.1 ± 9.2	23.4 ± 12.5	21.2 ± 11.6	0.001
DBIL, μmol/L	3.6 ± 2.2	5.3 ± 4.6	4.6 ± 3.8	0.001
Creatinine, μmol/L	62.1 ± 12.0	75.6 ± 23.0	70.0 ± 20.3	<0.001
BUN, mmol/L	4.7 ± 1.7	6.1 ± 2.3	5.5 ± 2.2	<0.001
eGFR, ml/min/m^2^	124.3 ± 28.2	93.4 ± 17.7	109.3 ± 23.2	<0.001
ESR, mm/h	3.0 (2.0–7.0)	2.0 (1.0–6.0)	2.0 (2.0–6.5)	0.527
CRP, mg/L	2.0 (1.5–3.4)	2.8 (1.9–4.2)	2.5 (1.7–3.8)	0.698
Cholesterol, mmol/L	4.0 ± 0.8	4.0 ± 0.9	4.0 ± 0.9	0.745
LDH, U/L	188 (161–219)	225 (192–270)	210 (180–255)	0.177
NT-proBNP, pg/mL	1,009.4 ± 790.9	1,510.0 ± 1,022.3	1,302.0 ± 963.3	<0.001
Uric acid, μmol/L	301.7 ± 51.6	503.8 ± 108.4	418.6 ± 129.6	<0.001
Digoxin, *n* (%)	74 (86.0)	108 (89.3)	182 (87.9)	0.485
Diuretics, *n* (%)	86 (100.0)	120 (99.2)	206 (99.5)	0.398
Warfarin, *n* (%)	71 (82.6)	97 (80.2)	168 (81.2)	0.664
Targeted Therapy^†^, *n* (%)	72 (83.7)	105 (86.8)	177 (85.5)	0.538
Monotherapy, *n* (%)	69 (80.2)	102 (84.3)	171 (82.6)	0.447
ERAs, *n* (%)	12 (14)	17 (14)	29 (14)	0.984
PDE-5 is, *n* (%)	55 (64.0)	77 (63.6)	132 (63.8)	0.963
Prostanoids, *n* (%)	2 (2.3)	8 (6.6)	10 (4.8)	0.156
Combined therapy, *n* (%)	3 (3.5)	3 (2.5)	6 (2.9)	0.670
ERAs + PDE-5 is, *n* (%)	3 (3.5)	3 (2.5)	6 (2.9)	0.670

*Values are expressed mean ± standard deviation or mean (interquartile range) or n%. BMI, body mass index; WHO FC, World Health Organization functional classes; RAP, right atrial pressure; mPAP, mean pulmonary artery pressure; PVR, pulmonary vascular resistance; CI, cardiac index; WBC, white blood cells; TBIL, total bilirubin; DBIL, direct bilirubin; BUN, blood urea nitrogen; eGFR, estimated glomerular filtration rate; ESR, erythrocyte sedimentation rate; CRP, C-reactive peptide; LDH, lactate dehydrogenase; NT-proBNP, N-terminal pro-B type natriuretic peptide; ERAs, endothelin receptor antagonists; PDE-5 is phosphodiesterase-5 inhibitors.*

† *At index hospitalization*.

### Baseline Serum UA Levels and Disease Severity

**Figure 1:**
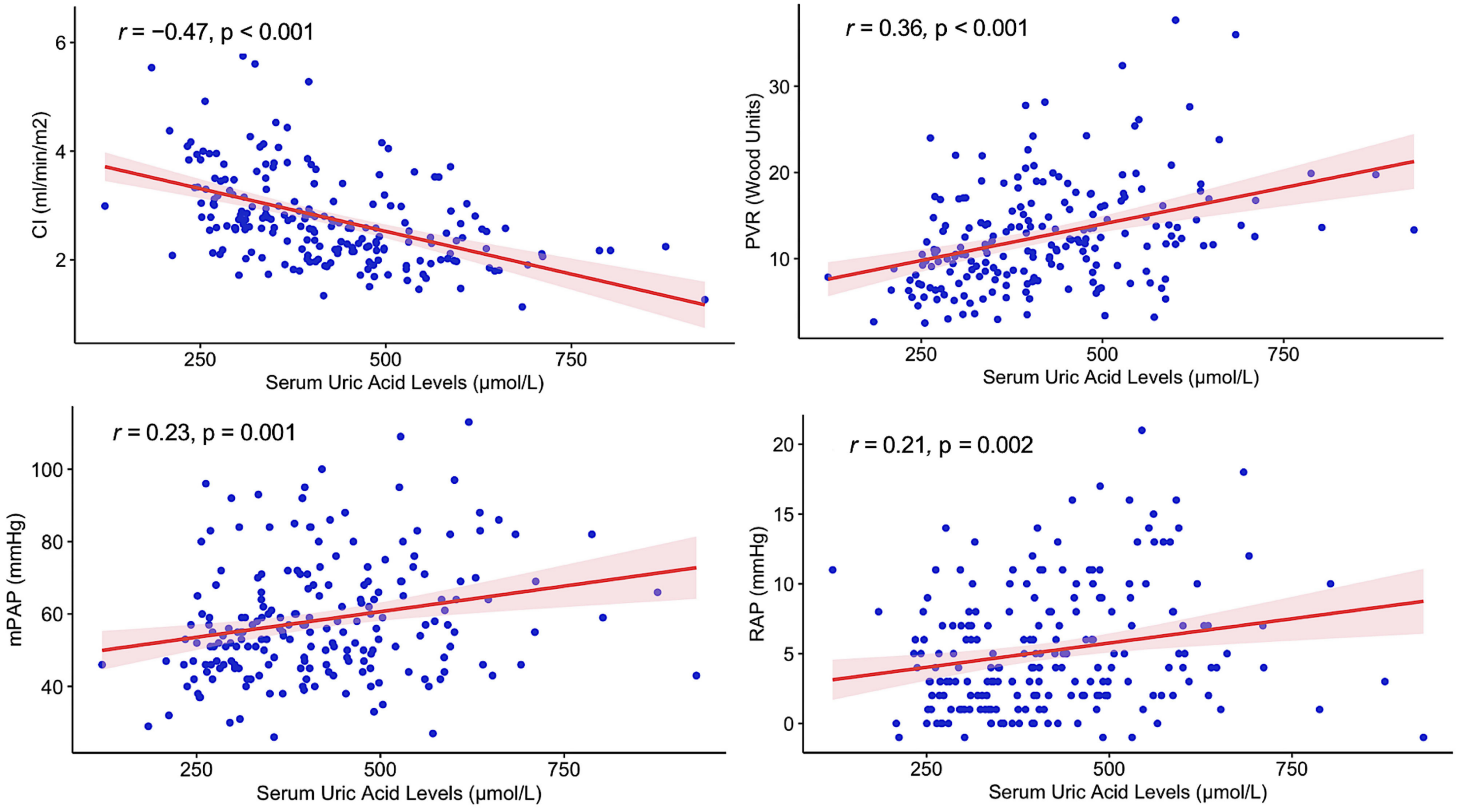
Correlations between serum uric acid levels and hemodynamic parameters in patients with idiopathic pulmonary artery hypertension. CI, cardiac index; PVR, pulmonary vascular resistance; mPAP, mean pulmonary artery pressure; RAP, right atrial pressure.

In Figure 1, Pearson's associations between baseline UA levels and major hemodynamic parameters at RHC were plotted. In patients with IPAH, serum UA levels positively correlated with PVR (*r* = 0.36, *p* < 0.001), mPAP (*r* = 0.23, *p* = 0.001), and RAP (*r* = 0.21, *p* = 0.002), and negatively correlated with cardiac index (*r* = −0.47, *p* < 0.001). Serum UA levels did not significantly correlate with age (*p* = 0.436) but were significantly associated with the cardiac index, NT-proBNP, creatinine, and BMI in univariate Pearson's correlation analysis (Table 2). In multivariate stepwise linear regression analysis, serum UA levels remained significantly associated with CI (β = −35.712, *p* = 0.005), BMI (β = 5.778, *p* = 0.010), creatinine (β = 3.354, *p* < 0.001), age (β = −2.801, *p* < 0.001), and NT-proBNP (β = 0.023, *p* = 0.004) (Table 2).

**Table 2:** Analysis of variables associated with serum uric acid levels in patients with idiopathic pulmonary artery hypertension.

**Variables**	**Pearson's correlation analysis**			**Multiple regression analysis**		
	***r***	**95% CI**	***P*-value**	**β**	**SE**	***P*-value**
RAP	0.212	0.078–0.339	0.002		
mPAP	0.231	0.098–0.356	0.001			
PVR	0.359	0.235–0.473	<0.001			
CI	−0.472	−0.572–−0.359	<0.001	−35.712	12.468	0.005
NT-proBNP	0.344	0.218–0.459	<0.001	0.023	0.008	0.004
Creatinine	0.588	0.492–0.671	<0.001	3.354	0.347	<0.001
Age	−0.054	−0.189–0.083	0.436	−2.801	0.677	<0.001
BMI	0.153	0.017–0.284	0.028	5.778	2.229	0.010

*CI, confidence interval; SE, standard error; RAP, right atrial pressure; mPAP, mean pulmonary artery pressure; PVR, pulmonary vascular resistance; CI, cardiac index; NT-proBNP, N-terminal pro-B type natriuretic peptide; BMI, body mass index*.

### Hyperuricemia, Serum UA Variability and 5-Year Mortality

Comparison of baseline serum UA levels in survivors and non-survivors demonstrated a significantly higher median UA than in non-survivors (464.6 vs. 411.6 μmol/L, *p* = 0.028). There were 32 deaths in the entire cohort over the study period, accounting for a 15.5% mortality rate. Twenty-six deaths occurred within the hyperuricemia group and six within normouricemia group (*p* = 0.003).

**Figure 2:**
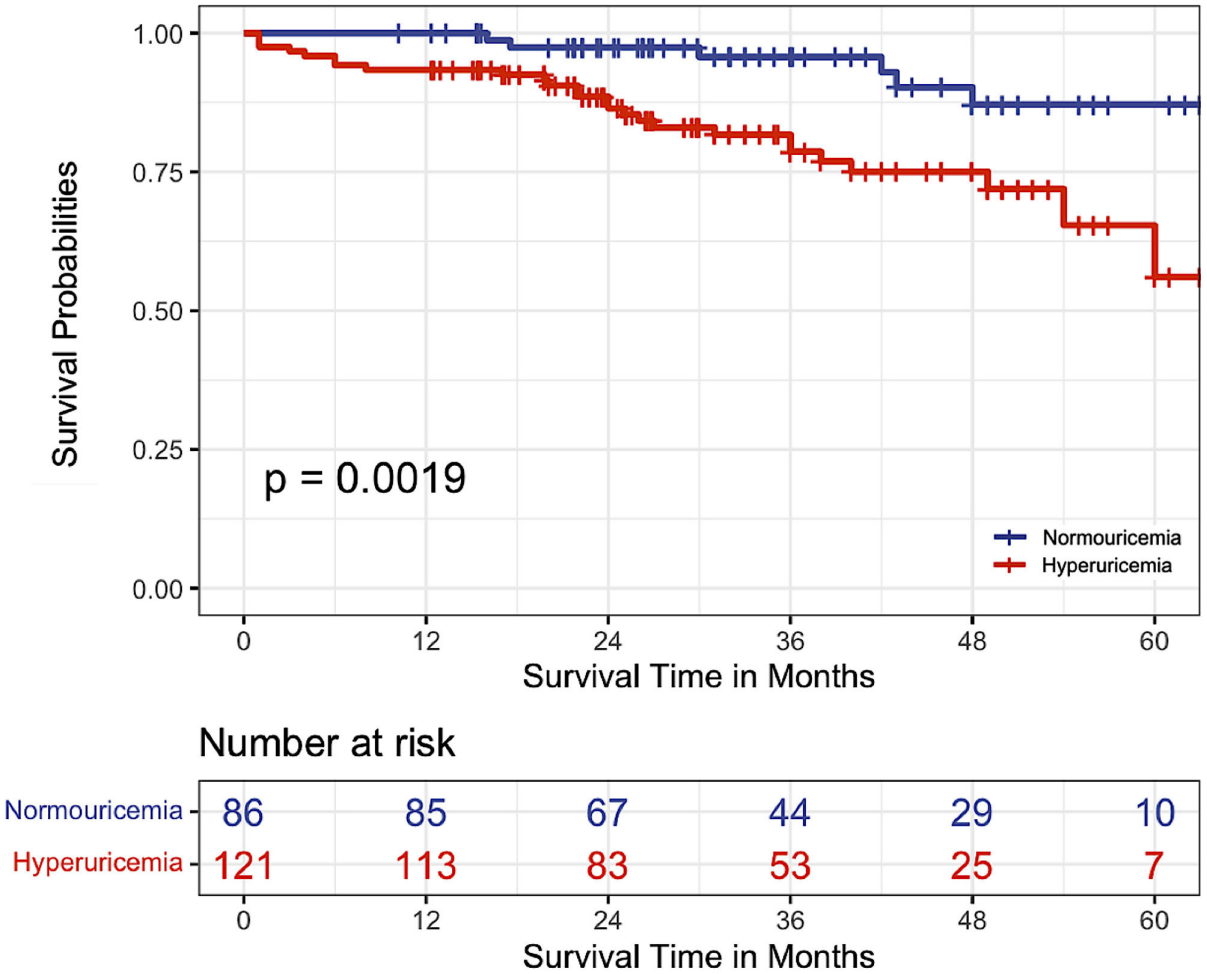
Kaplan-Meier survival curves according to the serum uric acid levels in patients with idiopathic pulmonary artery hypertension.

When Kaplan-Meier survivals curves were plotted according to hyperuricemia and normouricemia groups, elevated serum UA levels were associated with a higher mortality rate (log-rank *x*^2^ = 9.6, *p* = 0.002, Figure 2). The estimated 1-, 3-, and 5-year survival rates in IPAH patients with hyperuricemia were 93.4, 78.6, and 56%, respectively. On univariate Cox proportional hazard analysis, hyperuricemia was associated with a 3.73-fold increase in mortality [hazard ratio (HR) 3.73, 95% confidence interval (CI) 1.53–9.08, *p* = 0.003]. Multivariate Cox proportional hazard analysis included variables that were statistically, or clinically prognostic biomarkers related to mortality. After adjusting for age, gender, WHO functional classes, NT-proBNP, mPAP, absence of targeted therapy, and other hemodynamics, hyperuricemia also remained significantly associated with 5-year mortality (HR 2.61, 95% CI 1.02–6.68, *p* = 0.046) (Table 3).

**Table 3:** Multivariate cox proportional hazards analysis of variables associated with 5-year mortality in patients with idiopathic pulmonary artery hypertension.

**Variables**	**HR**	**95% CI**	***P*-value**
Age	1.009	0.979–1.041	0.559
Female gender	0.916	0.406–2.065	0.832
Absence of targeted therapy	2.092	0.568–7.715	0.267
WHO FC III-IV	1.414	0.603–3.314	0.425
Hyperuricemia	2.606	1.017–6.679	0.046
NT-proBNP	1.000	1.000–1.001	0.011
RAP	1.071	0.985–1.164	0.111
mPAP	1.016	0.979–1.054	0.401
PVR	0.981	0.898–1.072	0.676

*HR, hazard ratios; CI, confidence interval; WHO FC, World Health Organization functional classes; NT-proBNP, N-terminal pro-B type natriuretic peptide; RAP, right atrial pressure; mPAP, mean pulmonary artery pressure; PVR, pulmonary vascular resistance*.

**Figure 3:**
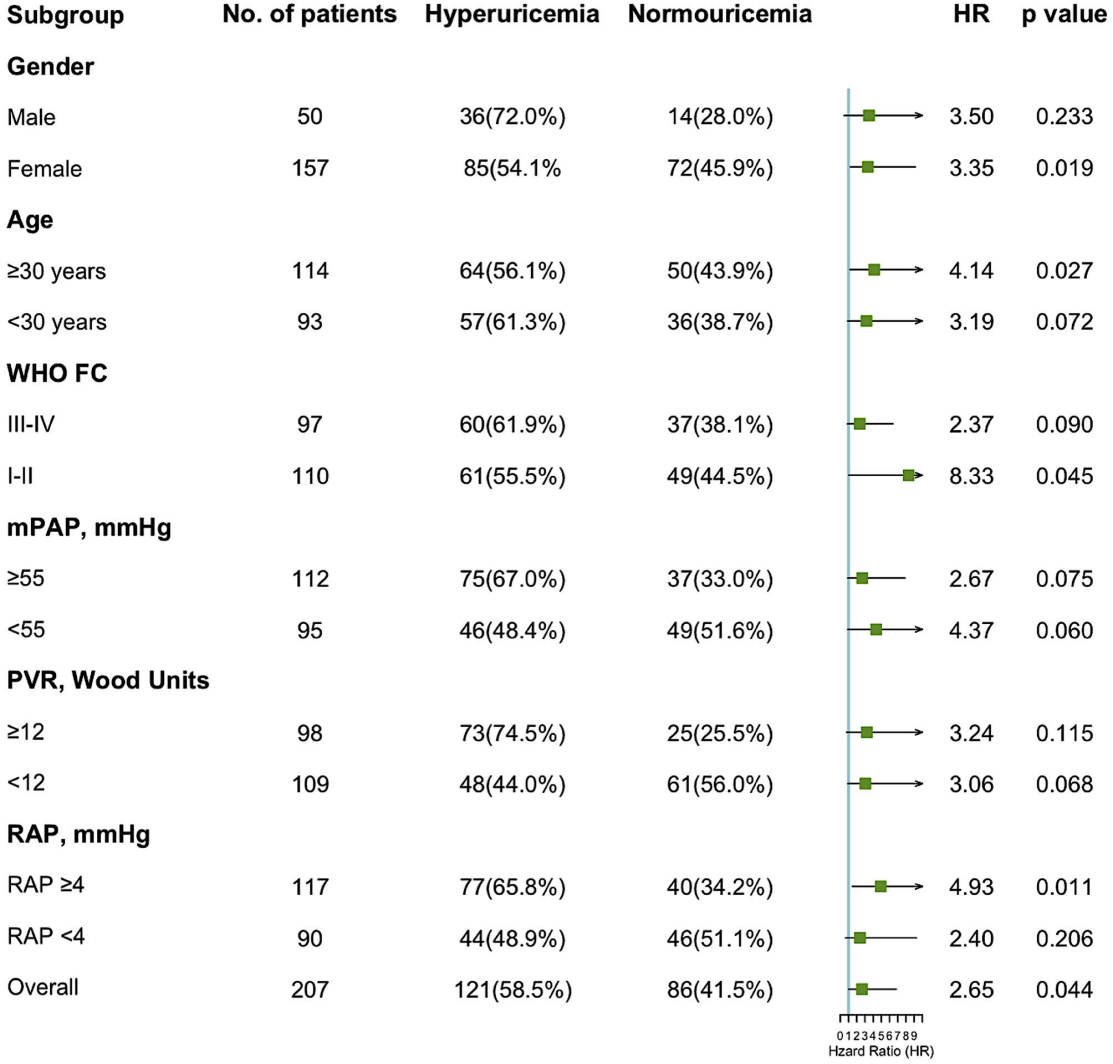
Forest plot of hazard ratios by patient subgroups. HR, hazard ratio; WHO FC, World Health Organization functional classes; mPAP, mean pulmonary artery pressure; PVR, pulmonary vascular resistance; RAP, right atrial pressure.

In stratified analyses according to gender, age, WHO functional classes, and severity of IPAH based on the median value of hemodynamic parameters, the impact of hyperuricemia on the hazard of mortality was plotted (Figure 3). The increase of the 5-year mortality rate with hyperuricemia was consistent across different subgroups, especially among females (HR 3.35, *p* = 0.019), patients aged ≥30 years (HR 3.19, *p* = 0.072), and patients with RAP ≥4 mmHg (HR 4.94, *p* = 0.011).

**Figure 4:**
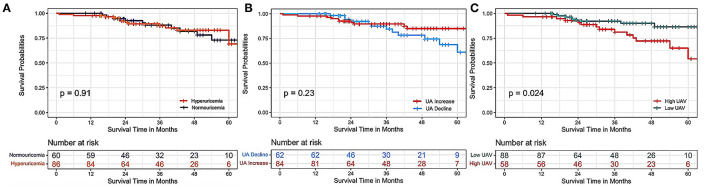
Kaplan–Meier survival curves according to serum UA levels at first follow-up and their variability with respect to baseline levels. **(A)** Kaplan–Meier survival curves of patients with hyperuricemia and normouricemia according to the uric acid levels re-evaluated at the first follow-up (*p* = 0.91). **(B)** Kaplan–Meier survival curves of patients with an increase or decline in serum UA levels at first follow-up compared with baseline UA levels (*p* = 0.23). **(C)** Kaplan–Meier survival curves of patients with a high or low variability of change in serum UA levels at first follow-up compared with baseline UA levels (*p* = 0.024). UAV, uric acid variability.

The second measure of serum UA (412.6 ± 126.7 μmol/L) at first follow-up was available in a subset of 150 patients. As shown in the Kaplan-Meier curves in Serum Uric Acid and Long-Term Mortality in IPAH Patients (Figure 4), there were no significant differences in the hyperuricemia and normouricemia groups at the first follow-up in predicting the 5-year death (log-rank test, *p* = 0.91). A changing tendency, i.e., an increase or decrease in UA levels, also yielded negative results (*p* = 0.23). Interestingly, IPAH patients with a high UAV, compared with those with low UAV, are associated with an increased all-cause mortality rate (*p* = 0.023).

## Discussion

In the present study, we investigated the utility of serum UA as a predictor of disease severity and long-term mortality in patients with IPAH. The main findings were that (1) a relatively high prevalence of hyperuricemia was observed in patients with IPAH (58.5%); (2) serum UA levels at baseline positively correlated with mPAP, RAP, and PVR, while negatively correlated with a cardiac index which remained significant after adjusting for potential confounders; (3) patients with hyperuricemia at baseline and those with high variability in serum UA at first follow-up had a significantly lower survival rate than those with normouricemia during follow-up of 5 years; and (4) hyperuricemia was independently associated with the 5-year mortality, and the association consisted in specific subgroups.

In the last guidelines for PAH risk stratification at baseline and during follow-up, the only two biomarkers that function as dynamic measures are the NT-proBNP and the brain natriuretic peptide (BNP) (11). Nevertheless, not all medical institutions, especially those in developing countries, have the opportunity to use this marker, and hence there is a clear need to explore new non-invasive biomarkers that could be routinely used in clinical practice. The utility of serum UA levels has been implicated in obesity, diabetes, coronary atherosclerosis, hypertrophic cardiomyopathy, left atrial thrombus in non-valvular thrombus, and other cardiovascular conditions (7, 13–15). Registries and clinical trials involving PAH indicated that the proportion of elderly patients at high cardiovascular risk increases progressively (16, 17). Elevated serum UA levels correlate with a higher rate of cardiovascular comorbidities and influence disease prognosis. The present study was consistent with prior studies showing diagnostic and prognostic use of serum UA in different forms of pulmonary hypertension (5, 8–10). Considering the gender-specific differences in UA levels due to estrogen-induced enhancement in renal rate elimination (18), we defined hyperuricemia and normouricemia according to the gender of patients. Our data showed that patients in the hyperuricemia group had more severe hemodynamic impairment (e.g., lower cardiac index) and long-term mortality in a large population of patients with IPAH, in contrast to what was previously described in other forms of PAH (8, 9) or a relatively small study sample (5, 10). Also, these observational trials have used varying definitions and techniques (echocardiography vs. RHC) for pulmonary hypertension, which may have further blurred the canvas. Notably, in accordance with literature established before the modern management of IPAH (10), we reinforce the relationship between higher UA levels and long-term mortality in the contemporary era. We demonstrated 2- to 3-fold higher hazard of death among hyperuricemia patients with IPAH and specific subgroups.

Among a range of hemodynamic parameters, the cardiac index remained the most vital related factor of serum UA levels, and our study revealed an inverse relationship between serum UA levels and cardiac index. Cardiac index (the body-surface-area-adjusted cardiac output) was recorded and analyzed to avoid potential influence by variant body weight and height. The fundamental basis accounting for this phenomenon is attributed mainly to lower perfusion to the kidneys, which compromises the renal excretion of UA. Meanwhile, reduced perfusion to the lungs with a low cardiac index may also result in hypoxia, and therefore, accumulation of serum UA (19). Additionally, we stratified patients into several groups according to the median value of demographic data and hemodynamic metrics indicative of pulmonary hypertension severity. The increase of mortality rate after a 5-year follow-up remained persistent and was, yet, more pronounced in patients aged ≥30 years and those with much severe PAH (indicated by RAP ≥4 mmHg). The latter shed new insights into different hazards of various subgroups. The recent meta-analysis has also concluded that hyperuricemia is a risk factor for subsequent or future pulmonary hypertension development and poor prognosis (6).

UA is a heterocyclic compound that is produced during the degradation of purines, which represents the end-product of purine metabolism in humans. The circulating concentration of serum UA results from a delicate balance between the catabolism of purines and their disposal via the kidneys. Purine products were ever identified as a closely correlated factor of right ventricular-pulmonary vascular dysfunction in patients with pulmonary hypertension (20). Serum UA is generated via oxidation action of the xanthine oxidoreductase (XOR) on xanthine, resulting in the production of reactive oxygen species (ROS) (21). Prior studies have illustrated that ROS could result in endothelial damage, intimal thickening, and fibroblast production, which are critical factors that may contribute to vascular injury in IPAH (22). In a rat model of hypoxia-induced pulmonary hypertension, XOR activity significantly increased during hypoxic exposure, and as rats treated with the XOR inhibitor allopurinol, pulmonary pressure, right ventricular hypertrophy, and pulmonary vascular remodeling were dramatically attenuated (23). Besides, *in vitro*, UA can also stimulate platelet-derived growth factors and vasoconstrictors such as endothelin-1 and angiotensin II (24, 25).

Further, experimental research suggests that UA and its downstream radicals may have pro-inflammatory, vasoconstrictive, and vascular remodeling effects (26). Precisely, it has been elucidated that oxonic acid-induced mild hyperuricemia in rat models is related to the development of renal small-vessel disease, characterized by the thickening of the pre-glomerular arteries and proliferation of smooth muscle cells (27, 28). The findings strongly indicate that UA may cause progressive kidney disease. Based upon these observations and the relationship between higher UA levels and the severity of PAH, we raised the question of whether and to what extent serum UA could exert a pathologic impact on the progression of PAH. Both vasoconstriction and enhanced accumulation of pulmonary artery smooth muscle cells (PA-SMCs) within the vascular wall contribute to the progressive course of pulmonary hypertension or PAH. Previous experimental studies also demonstrated that UA could stimulate smooth muscle cell proliferation by increasing angiotensin-II production, cyclooxygenase 2, endothelin-1, platelet-derived growth factor, ROS, and activating different pathways including extracellular signal-regulated protein kinases and p38 kinase pathway, NF-κB and c-Jun/AP-1 pathway (24, 29, 30). Further, in a recent *in vivo* experimental study, it was reported that local UA production is observed in the pulmonary vasculature of patients with IPAH, and higher UA concentrations could also promote cell growth and ROS production in PA-SMCs (31). Additionally, in two animal models of severe PH, local UA production is increased (31). In this regard, these findings could provide some plausible mechanisms that UA has a direct contributory and pathogenic role in the progression of PH, which could also impose devastating effects on the prognosis of patients with IPAH.

Previous studies revealed discrepant findings on the impact of various timepoints of serum UA levels on prognosis. Some supported that the higher baseline UA level would result in adverse outcomes such as all-cause mortality (32), while others emphasized the significance of UA levels at first follow-up (31) or persistent hyperuricemia (9). These inconsistencies may be due to a single measure of UA at baseline, different disease subtypes, and ethnic disparities in the study population. By contrast, in our current study, high UA at baseline and high variability from baseline to the first follow-up seemed to play a more fundamental role in predicting mortality risk. There are also potential biological mechanisms explaining why the variation of UA Levels matters more than the baseline levels. First, uric acid at baseline levels in treatment naïve patients may represent the disease burden at the initial stage of metabolism. Second, serum UA is regarded as a sign of metabolic changes related to oxidative stress, endothelial dysfunction, and activation of the renin-angiotensin system (21, 22). In this case, UA fluctuations may contribute to a pathological acceleration of these processes and thus aggravate health outcomes (33). Second, presumably, individuals with high variability of UA may carry significant risk factors for mortality such as dyslipidemia, smoking, and hypertension in some way. Third, a significant fall or rise in serum UA could stimulate and speed up urate crystallization, thereby stimulating one's immune and inflammatory responses (34). Further investigations are required to clarify the assumptions mentioned above.

Elevated levels of circulating UA at baseline were found to be significantly correlated with increased severity of IPAH based on invasive hemodynamic metrics, as well as increased risk of 5-year mortality. High variability in serum UA also confers risk on long-term survival. The study thereby highlights the importance of maintaining normouricemia, achieving stable serum UA levels, and avoiding large fluctuations. Exploring this easily measurable, non-invasive, and cost-effective biomarker would identify the susceptible high-risk patients who may benefit from timely and targeted therapeutic interventions. Evaluation of all prognostic information (e.g., baseline serum UA levels and UA variability) and therapy decisions should be made individually in patients with IPAH. Presumably, combination therapy that targets multiple signaling pathways with or without specific UA-lowering agents may be appropriate to reduce disease progression and mortality risk in IPAH patients with a high baseline UA or variability. This speculation would be more robust if serum UA levels significantly improved the risk discrimination power of the simplified risk table (11). However, the assumptions mentioned above need to be further confirmed by future large, prospective, and population-based studies.

The present study offers several advantages over previous literature regarding UA in IPAH. RHC, the gold standard of diagnosis of PAH, was performed in all recruited patients. We consolidated the evidence on the relationship between serum UA levels, particularly the high variability in UA levels and long-term mortality in a relatively large population of patients with IPAH in the contemporary era. Also, subgroups analysis was conducted to estimate the impact of hyperuricemia on mortality rate to recognize the high-risk group of patients. The potential limitations of this study also merit consideration. First, the nature of this retrospective cohort study would not allow us to avoid the possibility of selection bias. Second, the analysis on the UAV was limited to a subgroup of patients and therefore rendered a limited power to apply multivariate Cox proportional regression model. Third, given the observational study design of the present analysis, we cannot exclude the possibility of residual or unmeasured confounding. Last, the variability of serum UA may be affected by PAH treatment and UA-lowering therapies. However, further analyses showed no differences in the serum UA levels at the first follow-up between groups with and without PAH targeted drugs. Notably, the interaction effect on the uric acid levels at the first follow-up (*p* = 0.825) and UA variability (*p* = 0.435) between treatment groups and whether with or without treatment was also not statistically significant. Regarding the effects of UA-lowering agents that were not available and collected in our study, a meta-analysis of 35 randomized controlled trials in patients with gout showed that UA-lowering therapy did not reduce the major adverse cardiovascular events, including the all-cause mortality (35).

## Conclusion

In conclusion, baseline hyperuricemia and high variability in serum UA at first follow-up were related to a higher 5-year mortality in patients with IPAH. Routine assessment of serum UA levels may aid in severity assessment, risk stratification, and personalized treatment in patients with IPAH.

## Data Availability Statement

The raw data supporting the conclusions of this article will be made available by the authors, without undue reservation.

## Ethics Statement

The studies involving human participants were reviewed and approved by Fuwai Hospital Ethics Committee (No. 2018-1100). The patients/participants provided their written informed consent to participate in this study. Written informed consent was obtained from the individual(s) for the publication of any potentially identifiable images or data included in this article.

## Author Contributions

LY and ZH contributed to the study design, data analysis, and manuscript drafting and revision. ZZ and QZ critically reviewed and revised the manuscript. YT, YZ, XL, and AD performed literature search. QL and ZL provided professional advice on data interpretation, critically reviewed, and revised the manuscript. All authors contributed substantially to the work and agreed to submit the manuscript for publication.

## Funding

This study was funded by National Natural Science Foundation of China (81370326, 81641005, and 81800056), Beijing Municipal Science and Technology Project (Z181100001718200), National Precision Medical Research Program of China (2016YFC0905602), Double First-Class Discipline Construction Fund of Peking Union Medical College and Chinese Academy of Medical Sciences (2019E-XK04-02), CAMS Innovation Fund for Medical Sciences (CIFMS) (2020-I2M-C and T-B-055, 2021-I2M-C and T-B-032), Capital's Funds for Health Improvement and Research (2020-2-4033), and Beijing Municipal Natural Science Foundation (7202168).

## Conflict of Interest

The authors declare that the research was conducted in the absence of any commercial or financial relationships that could be construed as a potential conflict of interest.

## Publisher's Note

All claims expressed in this article are solely those of the authors and do not necessarily represent those of their affiliated organizations, or those of the publisher, the editors and the reviewers. Any product that may be evaluated in this article, or claim that may be made by its manufacturer, is not guaranteed or endorsed by the publisher.
